# Treatment of Neuropathic Pain Directly Due to Cancer: An Update

**DOI:** 10.3390/cancers14081992

**Published:** 2022-04-14

**Authors:** Morena Shkodra, Augusto Caraceni

**Affiliations:** 1 Palliative Care, Pain Therapy and Rehabilitation Unit, Fondazione IRCCS Istituto Nazionale dei Tumori, 20133 Milano, Italy; augusto.caraceni@istitutotumori.mi.it; 2 Institute of Clinical Medicine, University of Oslo, 0318 Oslo, Norway; 3 Department of Clinical Sciences and Community Health, Università degli Studi di Milano, 20122 Milano, Italy

**Keywords:** neuropathic cancer pain, opioids, antidepressants, anticonvulsants, analgesia

## Abstract

**Simple Summary:**

This review discusses treatment approaches for providing pain relief to oncological patients affected by pain caused by nerve damage due to the tumor, also known as neuropathic cancer pain. Although being encountered often and causing a relevant burden to these patients, neuropathic cancer pain remains still difficult to diagnose and treat. Strong evidence about the best drugs to be used remain limited, as do therapeutic choices.

**Abstract:**

Neuropathic pain can be defined as pain related to abnormal somatosensory processing in either the peripheral or central nervous system. In this review article, with neuropathic cancer pain (NCP), we refer to pain due to nervous tissue lesions caused by the tumor or its metastases. Nervous tissue damage is the cause of cancer pain in approximately 40% of those experiencing cancer pain. Recognizing a neuropathic pathophysiology in these cases may be difficult and requires specific criteria that are not homogenously applied in clinical practice. The management of this type of pain can be challenging, requiring the use of specific non-opioid adjuvant drugs. The majority of the criteria for NCP diagnosis and management have been based mainly on results from the noncancer population, risking the failure of addressing the specific needs of this population of patients. In this review, we summarize current management options available for NCP and provide some insights on new promising treatments.

## 1. Introduction

Neuropathic pain is defined by the International Association for the Study of Pain (IASP) Special Interest Group on Neuropathic Pain (NeuPSIG) as “pain arising as a direct consequence of a lesion or disease affecting the somatosensory system” [1].

There are many causes of neuropathic pain, through peripheral nerve lesions or brain and spinal cord lesions. The most common pathophysiological characteristic of pain due to neurological lesions is that the lesion affects the somatosensory pathways specifically involving the nociceptive fibers and their peripheral and central connectivity. Neuropathic pain in oncological patients can be caused by either the nerve damage from the cancer mass-effect and/or treatments including chemotherapy, radiotherapy, and surgery. However, in this article we refer to neuropathic cancer pain (NCP) as pain caused by the primary tumor or its metastases damaging or injuring the peripheral or central nervous system [2]. While the prevalence of pain due to neurological lesions in patients with advanced cancer can reach 40%, the neuropathic pathophysiology contributing to the individual pain experience is often unclear or at least not homogenously assessed according to individual clinical practices [3]. The local tumor’s direct effects and associated inflammatory processes are however always producing significant nociceptive mechanisms, which are often accounted for by considering a mixed, both nociceptive and neuropathic, pathophysiology in most cases of NCP pain [4,5].

The evaluation of the presence of NCP is relevant, since both its clinical characteristics and management are considered to differ from purely nociceptive and chronic neuropathic non-cancer pain [6]. These differences are partly related to the fact that NCP is often accompanied by a relevant nociceptive component but also to the fragility of oncological patients and the effect size of antalgic drugs used [7]. Clinically, neuropathic pain may be characterized by the presence of positive or negative sensory phenomena. NCP management is considered complex and often does not respond to opioid monotherapy, commonly requiring the use of adjuvant drugs such as antidepressants and anticonvulsants [8]. Due to its complexity, poor outcomes, and limited treatment options, NCP can become a relevant burden for patients, considerably impacting their quality of life [9].

Overall, there is little good clinical trial data on the use of adjuvant drugs and treatment approaches for NCP. This is, in part, related to the unique characteristics of these patients. The complexity and heterogeneity of pain characteristics, including the different clinical presentations of disease progression and the need for concomitant antineoplastic treatments, all can contribute to the difficulties of performing trials under stable experimental conditions. Additionally, relevant ethical concerns related to characteristics such as frailty require an accuracy in choosing the most appropriate designs and methodologies that take into account these specific characteristics. Existing treatment guidelines are highly dependent on trials in patients without cancer [10], failing often to address important issues such as side effects and the altered kinetics of these drugs in oncological patients [7]. Additionally, choice of treatments is often guided also by the coexistence of additional symptoms [9].

Despite challenges, progress in the understanding of the pathophysiology of neuropathic pain is spurring the development of new diagnostic procedures and personalized interventions, which emphasize the need for a multidisciplinary approach to the management of NCP. In this review article, we provide the state of the art in NCP treatment and novel promising therapeutic approaches developed.

## 2. General Principles

Opioid therapy is the first-line approach for moderate or severe chronic pain due to advanced cancer [11]. In some cases, however, additional analgesic interventions are considered [12]. In particular, it has been observed that NCP is associated with poorer outcomes when treated with only opioid analgesics [13], and analgesia can be improved by combining opioids with the so-called adjuvant medications [14]. Adjuvant analgesics commonly prescribed for NCP are the same as those used for chronic neuropathic pain of other causes [15].

Appropriate pain assessment is a key component for providing appropriate pain management [16]. Considering its heterogeneous characteristics, NCP diagnosis is not straightforward in most cases, and, to date, there is no accepted standardized gold standard for its assessment [4]. A grading system has been proposed by the Neuropathic Pain Special Interest Group (NeuPSIG) of the International Association for the Study of Pain (IASP), which can help identify probable or definite neuropathic pain in general [17]. The grading system is based on four criteria, as follows:

- Criterion 1: neuroanatomical plausible pain distribution;
- Criterion 2: suggestive history of a relevant neurological lesion or disease;
- Criterion 3: negative or positive sensory signs within innervation territory of the lesion;
- Criterion 4: confirmation of the lesion by a diagnostic test.

A probable diagnosis of neuropathic pain can be made if criteria 1, 2, and 3 or criteria 1, 2, and 4 are present. A definite neuropathic pain diagnosis is based on the presence of all four criteria. Yet, these criteria were not developed and validated for oncological patients; this is why in 2014, these criteria were revised and adapted for these patients, leading to the proposal of the EAPC/IASP algorithm for diagnostic criteria of NCP, which still needs validation [18]. 

Based on the association between pain characteristics, signs, and symptoms of the underlying lesion, NCP can be subdivided into specific pain syndromes according to the type of neurological involvement, including plexopathy, radiculopathy, and peripheral neuropathies, among other categories [19] (Table 1). Peripheral neurological lesions caused by the tumor are relatively frequent, while it is rare that neurological lesions due to cancer to the CNS cause pain, albeit in some cases of spinal cord lesions. The correct identification of the presence of NCP and its specific etiology can guide therapeutic decision making. For example, pain syndromes associated with focal, local, or regional pain can benefit from the application of topical treatments.

The diagnosis of a neuropathic component in the pathophysiology of pain should be made when cancer is causing a neurological lesion, and the pain is referred to the area of the neurological lesion of interest. Neurological negative signs and loss of motor function can confirm the presence of a neurological dysfunction, and pain can or cannot be associated with positive neurological signs of hyperexcitability, such as allodynia and hyperalgesia, that are typical of neuropathic pain. Sometimes, other symptoms, such as dysesthesia, paresthesia, burning or lancinating pain episodes, are also present and considered characteristic of NCP.

However, not all these clinical findings have been valued for establishing a diagnosis of neuropathic pain, especially in patients with cancer. Some screening questionnaires such as the DN4 (Douleur Neuropathique 4 Questions) [20], LANSS [21] (Leeds assessment of neuropathic symptoms and signs), and painDETECT [22] have been developed to identify the clinical characteristics of neuropathic pain and can be applied also to patients with cancer [5], but a homogenous and standardized method to combine clinical diagnosis and questionnaires results is still lacking. As a conclusion, the presence of a neurological lesion with signs and symptoms characteristic of neuropathic pain should be required for diagnosing NCP [23]. 

## 3. Pharmacological Management of NCP

Tricyclic antidepressants (TCAs), serotonin and norepinephrine reuptake inhibitors (SNRIs), and anticonvulsant drugs are effective in the management of neuropathic pain in general [24], and though data regarding their efficacy in NCP are scarce, they remain some of the main drugs used in these patients [25,26]. These drugs are commonly used in combination with non-opioid and opioid analgesics for NCP [26]. Due to the heterogeneity of patients and pain presentations, high-quality evidence to support the use of specific drugs for NCP is lacking; however, below we provide a list of some of the main drugs used for the pharmacological management of NCP.

### 3.1. Antidepressants

TCAs and SNRIs are commonly used for the treatment of NCP [26,27,28]. TCAs include drugs such as amitriptyline, nortriptyline, and desipramine; while SNRIs, which are often used for NCP, include venlafaxine and duloxetine [29]. The analgesic effect of antidepressants is independent of the antidepressant effect, and they exert this effect by reinforcing the descending inhibitory pathways, increasing the release of norepinephrine and serotonin in the synaptic cleft at both supraspinal and spinal levels [30]. A systematic review and meta-analysis on the effectiveness of antidepressants for NCP indicated that various trials reported a better response of combination therapy with antidepressants compared with treatments without antidepressants [8]. The absolute risk benefit (ARB) of antidepressants estimated in a review article based on five RCTs, three of which studied amitriptyline, was 0.55, and the absolute risk harm was0.13 [31]. 

SNRIs, mainly duloxetine, have been mostly studied and recommended for the treatment of chemotherapy-induced peripheral neuropathies [32]. However, a few recent studies have demonstrated that adding duloxetine to opioid or opioid–pregabalin therapy might be beneficial in patients with refractory NCP [33,34]. Additionally, a recent multicenter, randomized placebo-controlled trial performed at 12 specialized palliative care services in Japan, suggested that adding duloxetine to opioid–pregabalin therapy might have clinical benefit in alleviating refractory NCP [35]. Further studies are needed to conclude the efficacy of duloxetine in these cases.

Antidepressants can be helpful also in managing depression and anxiety accompanying chronic cancer pain. Yet, the potential benefits and risks should be well weighed before prescription. Drug toxicity can be dose-limiting for TCA, in particular, due to the anticholinergic effects. Common side effects of SNRIs include somnolence, dry mouth, dizziness, and increased sweating. 

### 3.2. Anticonvulsants

Neuronal hyperexcitability plays a relevant role in the pathophysiology of neuropathic pain. Gabapentinoids reduce pain transmission in the spinal pathways and also modulate the central descending inhibitory pathways and have, therefore, been widely used for the treatment of neuropathic pain [36]. In the systematic review of Jongen et al. [31], the ARB of the anticonvulsants was 0.57, and the ARH was 0.005. Gabapentin and pregabalin are two of the most frequently used drugs of this class for treating NCP. Both drugs have established efficacy for treating neuropathic pain and are also recommended as first line treatment for NCP [11,26]. In general, both gabapentin and pregabalin, can be effectively combined with opioids for this purpose. Somnolence and dizziness are the most frequent dose-limiting side effects. Although a few RCTs have shown the efficacy of these drugs when used for NCP [23,37,38], more data are needed on efficacy and safety outcomes in patients with NCP [39]. An open-label randomized study has investigated the use of pregabalin over fentanyl in patients with moderate-to-severe NCP, showing some benefit and suggesting that pregabalin monotherapy in NCP may lead to better control of the neuropathic component, with opioid-sparing effects [40]. Other antiepileptics include lamotrigine and second-generation anticonvulsants, carbamazepine and oxcarbazepine, which are however less commonly used for NCP [41,42,43,44].

### 3.3. Opioids

Since the publication of the World Health Organization (WHO) analgesic ladder in 1986 [45], opioids have remained the mainstay therapy in cancer patients experiencing moderate to severe pain [11]. Considering the particular nature of cancer pain, the presence of a nociceptive component is very common, also in patients with NCP, making it difficult to objectively determine the effect of the analgesics on the neuropathic component. [46]. For this reason, control of NCP usually requires the concurrent use of opioids and adjuvant analgesics specific for NCP [26,47]. A systematic review and meta-analysis on the combined use of opioids with antidepressants or anticonvulsants indicated a nonsignificant improvement of pain relief in patients with NCP compared with opioid monotherapy, but the heterogeneity of the pain conditions in the studies did not allow the formulation of firm conclusions [48]. A balance should always be made between the benefits in terms of pain intensity improvement and the increased risk of adverse effects due to the combination therapy in these patients.

Strong opioids, including drugs such as morphine, oxycodone, methadone, and fentanyl, are used in moderate or severe cancer pain but also NCP and neuropathic pain from other chronic nonmalignant conditions [49]. Methadone is a synthetic opioid-receptor agonist and N-methyl-D-aspartate (NMDA) inhibitor [50]. A recent prospective cohort pilot study on patients with NCP indicated that methadone could significantly improve neuropathic pain through a targeted effect on allodynia and the pressure/squeezing component [51]. Due to its unpredictable very long half-life and risk of accumulation and toxicity, its use should be reserved to experienced clinicians [11,26].

Regarding drugs used for moderate-intensity pain, tramadol deserves some attention, since it is a centrally acting drug, with both opioid activity and monoaminergic properties, and has been used in the treatment of neuropathic pain [52]. A double-blind placebo-controlled study on patients with NCP has shown that tramadol was significantly more effective than the placebo in improving pain intensity and quality of life, decreasing the use of antiepileptic analgesics [53]. However, to date, there is still only modest evidence about the effectiveness of tramadol in NCP [54]. Tapentadol is a dual-acting μ-opioid receptor agonist and noradrenaline reuptake inhibitor, and its use has been suggested also for NCP, especially for cases in which an immediate dose adjustment is required or in patients at high risk for adverse effects [55,56].

Sometimes, the type of opioid or the route of its administration needs to be changed, either due to the presence of significant side effects or lack of response. This may be the case also for patients affected with NCP. The substitution of one opioid with another is also known as opioid switching or rotation [57] and has been found to improve opioid responsiveness [58]. The existence of an incomplete cross-tolerance among opioid agonists, genetic polymorphisms, and interindividual variations can partly explain the clinical improvement obtained in these cases [59]. The achievement of satisfactory pain control and decrease in adverse effects intensity after opioid rotation has been reported in around 50–90% of patients [60]. Particularly, methadone has been shown to improve pain control and reduce opioid toxicity in cancer patients who are receiving treatment with high doses of other opioids and in cases of NCP presence [61]. Opioid switching requires, however, clinical experience and adequate monitoring, especially in patients receiving high dosages. Further research is needed to improve the knowledge about the conversion ratios in the different clinical situations.

### 3.4. Local Anesthetics

Topical lidocaine is a local anesthetic used also for cancer related pain. A topical lidocaine 5% patch has been shown to effectively treat postherpetic neuralgia and other localized neuropathic pain associated with the presence of allodynia [62,63,64,65]. A prospective nonrandomized open-label study of patients with pain post-thoracotomy and post-mastectomy or pain caused by chest wall tumors demonstrated that the addition of lidocaine 5% patches was effective in the short term for the treatment of neuropathic pain accompanied by allodynia, whether deriving from a painful scar or chest wall tumor [66]. However, these findings need to be confirmed by randomized controlled trials with larger samples and specific for NCP only. Additionally, it should be kept in mind that lidocaine is effective only for well localized pain, which is not common. Though systemic absorption of topical lidocaine is minimal, it should not be used in patients on class I antiarrhythmics [41].

### 3.5. NMDA Antagonists

NMDA is a receptor for the excitatory neurotransmitter glutamate, which is released with noxious peripheral stimuli. The activation of these receptors has been associated with hyperalgesia, neuropathic pain, and reduced functionality of opioid receptors [67]. Therefore, NMDA antagonists could play a role in the management of neuropathic pain. There are several NMDA receptor antagonists available, including ketamine and methadone. They each differ in their level of activity on the NMDA receptor. Ketamine is a strong NMDA antagonist, usually less tolerated than the other antagonists due to a higher incidence of side effects, in particular hallucinations and a dissociative mental state [68]. Although ketamine has been used as an adjuvant drug in challenging cancer pain [69], recent trials have failed to indicate any benefit from the use of the drug in treating NCP [70,71]. There are currently not enough data to recommend the use of ketamine for NCP, but it has been hypothesized that some subgroups of patients, such as those with central sensitization, could benefit from the use of this drug [71].

### 3.6. Cannabis

The endocannabinoid system is involved in many physiological functions and homeostasis. Cannabinoid receptors are expressed in the peripheral and central nervous system and on immune cells, therefore being potential targets for the modulation of pain processing. Cannabis is, in general, seen as an alternative to conventional treatment when patients do not respond or have severe side effects. Some evidence suggests that medical cannabis has the potential to effectively manage pain in cancer pain patients, and preclinical data in NCP models have suggested a potential analgesic role for cannabinoids [72,73]. However, although there are some positive results in pain of cancer patients, the clinical evidence for cannabinoids as analgesics is not yet demonstrated, and their use can only be weakly recommended [74]. A recent systematic review and meta-analysis to analyze the effects on pain and adverse effects of cannabinoids compared with a placebo or other active agents for the treatment of cancer-related pain showed that the addition of cannabinoids to opioids did not reduce cancer pain [75]. The efficacy of cannabinoids remains to be elucidated [76].

### 3.7. Interventional Treatment Options

Uncontrolled NCP can sometimes require the use of interventional analgesic techniques. Neuraxial analgesia consists in the delivery of local anesthetics, opioids, or coanalgesics into the epidural or intrathecal (spinal) space [77]. Intrathecal administration of morphine and other drugs such as bupivacaine or clonidine may provide better pain relief [78,79]. Clonidine is an alpha2-adrenergic agonist and has been approved for interventional administration in the treatment of neuropathic cancer pain [80]. Intrathecal ziconotide, a neuron-specific calcium channel blocker, is also an approved analgesic, which has been shown to provide pain intensity reduction in RCTs in noncancer and cancer pain patients [81,82]. Yet, these techniques require specialized equipment and a pain specialist, and although generally safe, they can be accompanied by relevant side effects [77]. The role of nerve blocks and neurolytic procedure in NCP is poorly documented.

### 3.8. Proposed Treatment Algorithm

**Figure 1:**
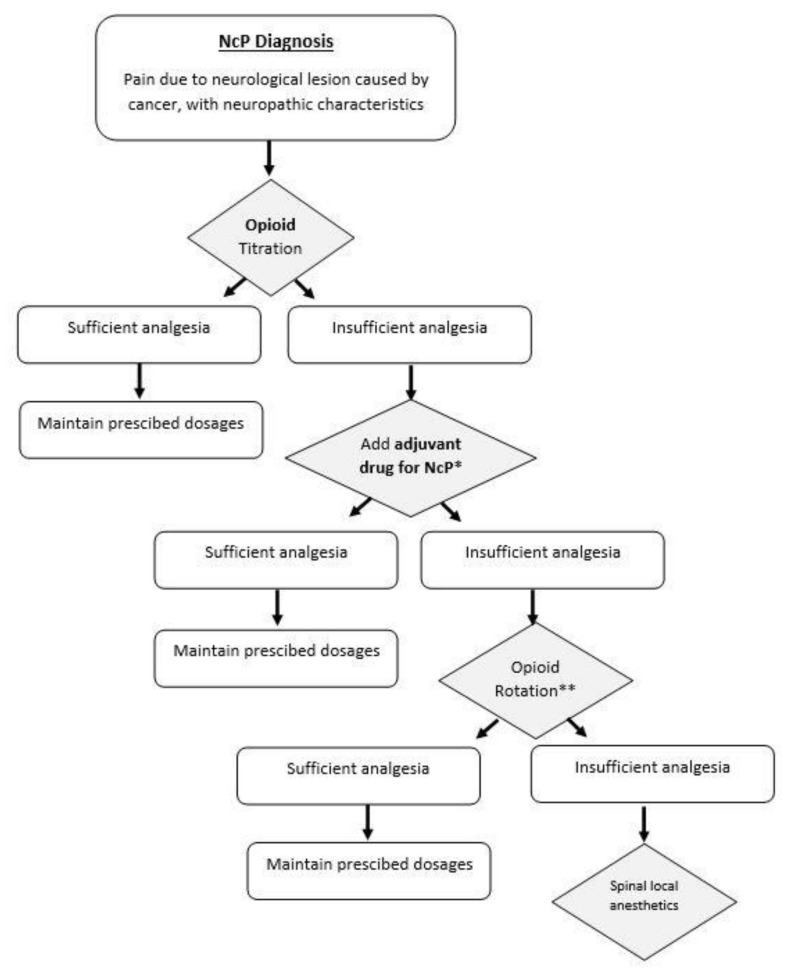
Neuropathic Cancer Pain (NcP) treatment algorithm based on clinical experience. * There is some evidence suggesting the benefit of adjuvant drugs such as gabapentinoids (gabapentin and pregabalin) and antidepressants such as amitriptyline. ** most commonly methadone is used, although requiring caution regarding its titration and dose conversion.

The pharmacological strategy for NCP treatment when analgesia is not satisfactory with the use of opioid medications is only weakly supported by clinical trials, and robust evidence is lacking. Most available studies have low quality of evidence often with unclear randomization procedures and heterogeneity, which make the pooling of the results not possible [83]. Based on the existing guidelines, but also on clinical experience, in our clinical practice the first step in for an appropriate management of NCP is the correct identification of this pain type, according to the pre-existing mentioned criteria [17]. After diagnosis of NCP, titration of monotherapy with opioids is attempted and maintained if it provides sufficient analgesia. In the case of insufficient analgesia and the coexistence of sensory symptoms, adjuvant drugs for NCP can be prescribed. Current evidence suggests benefits mainly using adjuvant drugs such as gabapentinoids, mainly gabapentin and pregabalin, and antidepressants such as amitriptyline [23,38]. The combination therapy is maintained if it provides adequate analgesia; otherwise, opioid rotation can be attempted. Methadone is usually the drug of choice although requiring cautions about its titration and dose conversion [60]. If pain persists, additional pain management approaches can be implemented, such as local spinal anesthetics. These steps have been summarized in Figure 1.

## 4. Novel Therapeutic Agents

Thanks to preclinical research studies, several advances have been made in better understanding the pathophysiology and neurobiology of pain in general. We provide here a summary of these findings and the drugs recently included in clinical practice or currently under investigation, for which positive effects in NCP treatment have been suggested either clinically or preclinically.

### 4.1. Tetrodotoxin

Tetrodotoxin (TTX) is a potent neurotoxin found mainly in puffer fish and other animals. It blocks voltage-gated sodium channels playing a key role in pain signaling [84]. During pathological pain conditions, such as neuropathic pain, upregulation of some TTX-sensitive voltage-gated sodium channels contribute to painful hypersensitization [85]. TTX displays a prominent analgesic effect in several models of neuropathic pain in rodents but is almost unexplored in preclinical models of pain induced by cancer, with only one article evaluating the role of TTX in bone cancer pain [86]. Using an in vivo behavioral test, they found that blockade of the dorsal root ganglion (DRG) neuron activity by intrathecal injection of TTX (10 μg/kg, once a day) inhibited the tumor-evoked mechanical allodynia and thermal hyperalgesia in bone cancer in rats. TTX has been tested in several clinical trials, including phase II and phase III trials. These studies have indicated promising results in terms of both the safety and efficacy of TTX in relieving pain associated with cancer [87]. A Phase IIa open-label study found that the 30 μg b.i.d. dose of TTX administered intramuscularly for 4 days appeared to be safe and efficacious in cancer patients whose pain was not relieved with standard therapy [88]. The role of TTX in cancer-related pain has been subsequently assessed in a larger Phase II randomized, multicenter, double-blind, placebo-controlled trial, which showed a statistically nonsignificant trend toward more responders in the active treatment arm based on pain intensity difference. A recent multicenter, randomized, double-blind, placebo-controlled, parallel-design trial on TTX (30 μg) given subcutaneously twice daily for four days, suggested a favorable benefit-risk profile and clinically important analgesic effect (Number Needed to Treat about 4–6) in the treatment of uncontrolled moderate to severe cancer-related pain [89]. However, this trial was underpowered, and more studies are needed to replicate and confirm the previous findings.

### 4.2. Botulinum Toxin Type A (BoNT-A)

BoNT-A is a potent neurotoxin produced by Clostridium botulinum, which mainly acts at the muscular level by inhibiting the release of acetylcholine at presynaptic levels, blocking the action potential in the neuromuscular junction [90]. In recent years, many experiments have been carried out in animal models but also on humans, investigating the role of BoNT-A in as an analgesic drug in both nociceptive and neuropathic cancer pain. Although its mechanism is not yet fully understood, evidence has shown that BoNT-A inhibits the secretion of pain mediators from the nerve endings and dorsal root ganglion, impacting directly on the nociceptive transmission through the anterolateral and trigeminothalamic systems [24,91].

### 4.3. TRPM8 Activator Menthol

Findings from basic science studies have shown upregulation of receptors for cooling in neuropathic pain models, indicating a role of cooling-induced analgesia as novel target for intervention [92,93]. Indeed, preclinical evidence has shown that the activation of the transient receptor potential melastatin 8 (TRPM8) ion channel, by topical agents, produces significant analgesia [94]. Clinical evidence also suggests that topical menthol, a TRPM8 activator, has potential as a novel analgesic therapy for localized cancer-related neuropathic pain, improving patient-rated measures [95].

### 4.4. Growth Factors Inhibitors

Growth factors, such as nerve growth factor, brain-derived neurotrophic factor, platelet-derived growth factor, insulin-like growth factor 1, and their respective receptors, have been identified as targets in the treatment of neuropathic pain [96]. Preliminary data have shown that Tanezumab, a monoclonal antibody against nerve growth factor can improve metastatic cancer-related bone pain [97,98]. However, its role in the management of NCP remains still to be elucidated. Recently, the epidermal growth factor receptor (EGFR) has also been identified as a potential therapeutic target. EGFR plays an important role in controlling functions such as growth, proliferation, metabolism, and survival, and, as such, its multifunctioning can drive tumorigenesis and tumor progression [99]. Anticancer drugs, which target EGFR, have been developed and are used for cancer treatment. However, recent studies and description of clinical cases have indicated that EGFR might be involved in pain and NCP pain treatment [100,101,102,103]. A recent proof-of-concept evaluation of an EGFR inhibitor used for neuropathic pain treatment did not provide statistical evidence of efficacy, although it demonstrated substantial reduction in pain [104]. These findings indicate that there might be a beneficial role of these drugs for NCP, which warrants further investigation.

### 4.5. Lemairamin

Lemairamin, isolated from the pericarps of the Zanthoxylum plants, is an agonist of α7 nicotinic acetylcholine receptors (α7nAChRs), which can reduce neuroinflammation in Alzheimer’s disease [105]. A recent study has evaluated its antinociceptive effects in pain hypersensitivity and explored the underlying mechanisms, showing that lemairamin could produce antinociception in pain hypersensitivity through the spinal IL-10/β-endorphin pathway following α7nAChR activation [106].

Other additional promising compounds can be found, which have shown some efficacy in pre-clinical studies and warrant further research. We might mention here drugs such as thalidomide targeting the IL-10/β-endorphin signaling pathway [107] or protopanaxadiol targeting the spinal microglial dynorphin A expression following glucocorticoid receptor activation [108].

## 5. Future Directions

There remain many challenges in the topic of NCP management, which open numerous opportunities for future clinical and preclinical research. Current guidelines on NCP are based on scarce evidence, which is often contradictory. It is clear that, in the future, more robust RCT are needed in order to obtain stronger evidence on existing recommendation. Good research is, however, largely based on good clinical practice and appropriate pain assessment; this is why, the identification of a gold standard for NCP diagnosis is imperative. Preclinical studies on the discovery of novel mechanisms and drugs are also needed. The very complex nature of cancer pain indicates that better insights are needed both regarding the neurobiology of pain in these patients and also on mechanisms and targeted therapies.

## Figures and Tables

**Table 1:** Pain syndromes due to nervous tissue lesions.

**1. Peripheral nerve syndrome**	a. due to paraspinal mass b. due to chest wall mass c. due to retroperitoneal mass other than paraspinal d. due to other soft-tissue or bony tumor e. peripheral polyneuropathy
**2. Radiculopathy or cauda equina syndrome**	a. due to vertebral lesion b. due to leptomeningeal metastases c. due to other intraspinal neoplasm
**3. Plexopathy**	a. cervical plexopathy b. brachial plexopathy c. lumbosacral plexopathy d. sacral plexopathy
**4. Cranial neuropathy**	a. due to base of the skull tumor b. due to leptomeningeal metastases c. due to other soft-tissue or bony cranial tumor
**5. Pain due to central nervous system lesion**	a. due to myelopathy b. intracerebral lesion
